# Liver cancer treatment with integration of laser emission and microwave irradiation with the aid of gold nanoparticles

**DOI:** 10.1038/s41598-022-13420-w

**Published:** 2022-06-03

**Authors:** Saeedeh Kabiri, Fatemeh Rezaei

**Affiliations:** 1 Department of Physics, Sharif University of technology, Tehran, 11155-9161 Iran; 2 Department of Physics, K. N. Toosi University of Technology, Shariati, Tehran, 15875-4416 Iran

**Keywords:** Biophysics, Computational biophysics, Nanoscale biophysics

## Abstract

This paper studies the effectiveness of the integration of microwave field irradiation and laser emission in liver cancer therapy with the aid of gold nanorods, in order to find out the influences of these combinational methods in tumor necrosis. Hepatocellular carcinoma is a kind of liver cancer that usually has a complicated structure, including both of superficial and deep sections. In current research, in deep regions of cancerous tissue, microwave antenna is utilized and in superficial regions, laser beams are irradiated. A Pulsed laser with heating time of 50 s and cooling time of 20 s is utilized for hyperthermia treatment. It should be mentioned that gold nanorods are injected into the tumorous region to enhance the treatment process and reduce the patient’s exposure time. Simulation results showed that at the first step, without any injection of gold nanoparticles, 0.17% of the tumor’s volume encountered necrosis, while at the next stage, after injection of gold nanorods, the necrosis rate increased to 35%, which demonstrates the efficiency of gold nanorods injection on the tumor treatments. Furthermore, the combinational applying of both microwave antenna and laser illumination can eradiate the tumor tissue completely.

## Introduction

According to world health organization (WHO) report in 2019, cancer is the first or second leading reason of death before the age of seventy in 91 countries among 112 countries. A common malignant neoplasm in worldwide is liver tumor which is the third most common cause of death in 2020 i.e. 830,000 deaths in world^1^. Liver cancer is one of the most lethal cancers because of its low ratio of recurrence rate, and high invasiveness. There are various methods for liver cancer treatment; such as: radiation therapy, chemotherapy, surgery, and laser (or photo) therapy. Considering the increase of cancer incidence around the world and the side effects of current treatment methods (such as pain, sleep problems, hair loss, and bleeding) it seems necessary to provide new therapeutic methods and substitute the old techniques by the new ones.

Some of the treatment methods mentioned above are based on hyperthermia mechanism, in which a high amount of heat is delivered to the tumor so that the tumor’s temperature increases upper than a specific threshold, usually between 39.5 and 40.5 °C, which cause cancerous cells’ death due to enzymatic changes^2^. There are different physical methods for heating up of the cancerous tissue, including microwave electric field, ultrasound irradiation, and photothermal therapy. The effectiveness of these methods is determined by the temperature achieved during treatment, the treatment duration, and the characteristics of the involved organ’s tissue^2,3^. One of the important challenges in hyperthermia is concentration of the adequate heat on a particular region, which is very important because it may cause extreme damages to the surrounding healthy tissues^4,5^. Photothermal therapy is a very localized treatment due to exploiting highly focused laser beam, while it is not a good choice for deep bulky tumors. It should be mentioned that microwave coagulation therapy is a very effective technique for coagulation of deep stiff tumors, while unwanted damages of healthy tissues is inevitable when it is used in superficial tumors.

Generally, addition of nanoparticles into different cancer therapies can improve the treatment process considerably. It should be stressed that nanoparticles and comprising nano-drugs are very impressive in destroying the cancerous cells with increasing the tumor temperature or decreasing the damage to the surrounding tissues^6,7^. Today, the use of mathematical modeling in treating cancer has become widespread and progressing, in parallel with the experimental studies. Mathematical modeling performs cost-free and in relatively low time, simulates the complex systems process by taking into account the conditions associated with the physical properties of the biological tissue, without any influences of the test on the human body, animal species or any biological tissues.

Several researchers have worked on liver cancer treatment by hyperthermia techniques based on laser irradiation with the aid of different nanoparticles^8–13^. For instance, Liu et al.^14^ have studied the usage of gold nanoshells functionalized with a small peptide in photothermal treatment of hepatocarcinoma. The functionalized gold nanoshells have illustrated good targeting performance in liver tumor cells BEL-7404 and BEL-7402 with a low cytotoxic activity, but they are not effective for the normal healthy liver cell HL-7702. Furthermore, their fluorescence images have presented that the gold nanoshells could induce the death of the liver cancer cells during in vitro experiments after treatment with a NIR laser irradiation. Sun et al.^10^ have used a semiconducting polymer for orthotopic liver cancer therapy during laser irradiation. They have shown that a 1064 nm laser, in comparison with 808 nm laser, causes more effective inhibition of orthotopic liver cancer cell growth in similar conditions. Furthermore, Iancu et al.^11^ have proposed a method based on multi-walled carbon nanotube (MWCNT) carrier system for increasing the laser thermal ablation of HepG2 cells (i.e. human hepatocellular liver carcinoma cell line). They have presented that human serum albumin-MWCNTs selectively attach to the albondin (aka Gp60) receptor in HepG2 membrane, accompanied by an uptake through a caveolin-dependent endocytosis occurrence. Moreover, different research groups have used the electric field pulses in combination with nanoparticle injection for liver cancer therapy^15^. For example, Minbashi et al.^16^ have reported the influences of the input power of a microwave antenna (MWAN) on hepatocellular carcinoma (HCC) tumor which was injected by magnetic nanoparticles (MNPs). First, they have used microwave antenna at a frequency of 2.45 GHz with a power of 90 W for 3-min ablation. Then, they have investigated the effects of different input power of MWAN after injection of MNPs. They have shown that the optimized simulated magnitudes of the external magnetic field and the input microwave power are 15 mT and 35 W, respectively. Moreover, chen et al.^17^ have treated hepatocellular carcinoma by mitochondria-targeting zirconia (ZrO_2_) complex nanoparticles (MZCNs) during microwave irradiation. They have shown that in case of in vivo experiment, the mice injected with MZCNs had an effective area with a temperature above 42 °C approximately 2.8-fold greater than the controls groups because of the targeting effect and higher microwave sensitivity of the MZCNs.

This paper is trying to investigate the effects of treatment of liver cancer by combination of the non-invasive microwave electric fields and nanoparticle-mediated laser photothermal therapy^8^. Due to complicated structure of Hepatocellular carcinoma, the mentioned combinational method is chosen for the first time in current research to reduce the probable side effects. Here, the results are obtained by theoretical and mathematical calculations based on the finite elements method. In the present work, a simulation solution is obtained by solving the bioheat transfer equation in the tissue during the liver cancer’s treatments.

## Materials and methods: physical problem and mathematical equation

Generally, cell death in thermal therapy may occur due to different mechanisms of necrosis, apoptosis, or autophagy. The heat generation can occur due to ultrasound irradiation or electromagnetic wave emission which comprises different wavelengths ranges of microwave, radiofrequency, and near-infrared^18–20^.

**Figure 1:**
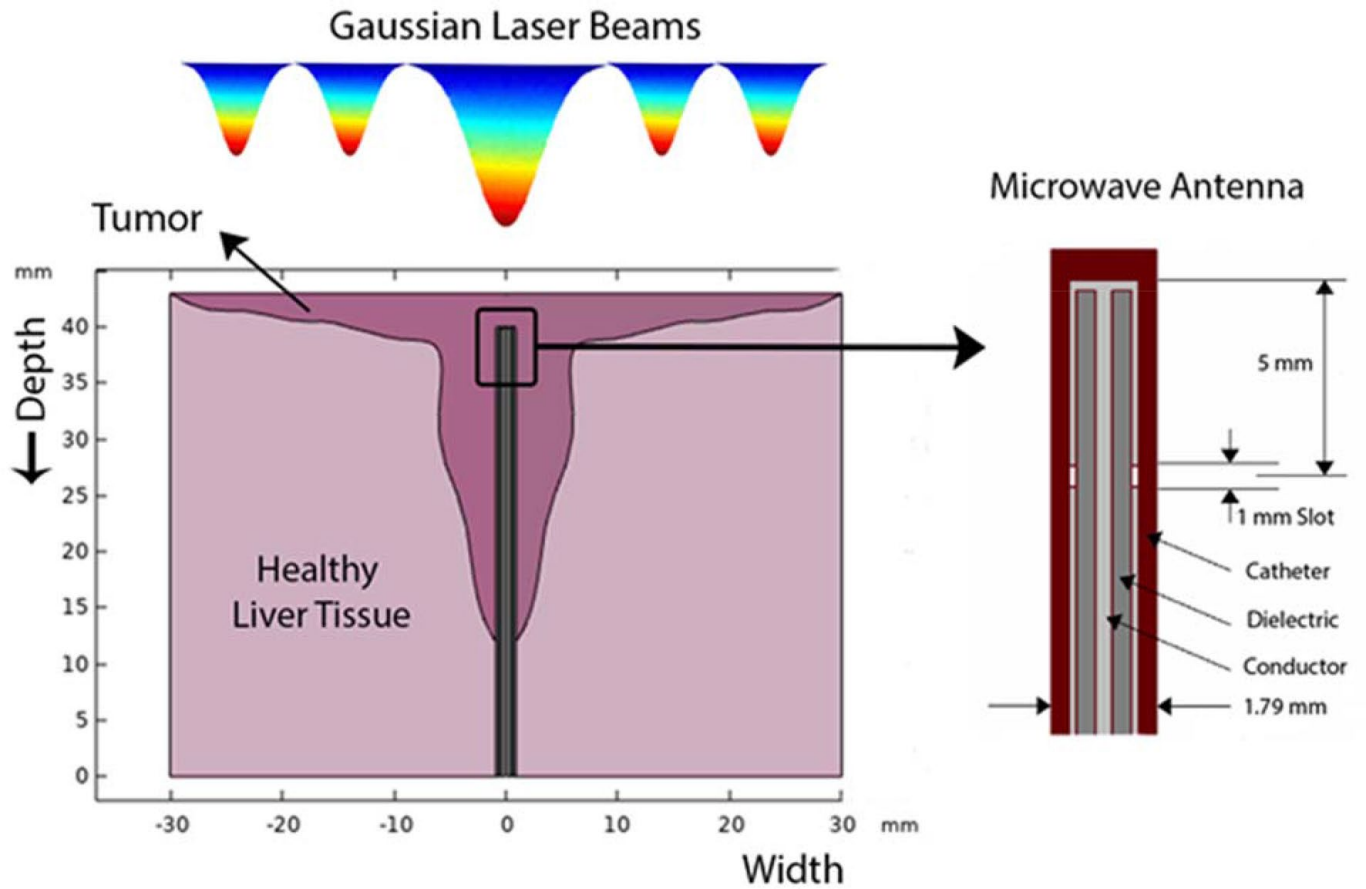
A typical Hepatocellular carcinoma exposure to both of electric field and laser irradiation.

In this study, the necrosis process of a liver tumor via combined usage of microwave irradiation and laser photothermal therapy is investigated. Figure 1 shows a schematic diagram of the tumor shape and the places where microwave irradiation and external collimated laser beam are implemented. The tumor is chosen to have an arbitrary shape including variable thicknesses of zero in superficial parts to 31 mm in deep regions, which comprises the diameter of 60 mm. Tables 1 and 2 show the physical properties of the tissues and blood, respectively.

**Table 1:** Thermal and optical properties of liver (healthy and cancerous tissue).

Property	Healthy tissue	Tumor
Density, $\rho$	1079 $[{\text{kg}}/{\text{m}}^{3} ]$^21^	1079 $[{\text{kg}}/{\text{m}}^{3} ]$^21^
Thermal Conductivity, $\kappa$	0.52 $[{\text{W}}/{\text{m}}.{}^{ \circ }{\text{K}}]$^21^	0.52 $[{\text{W}}/{\text{m}}.{}^{ \circ }{\text{K}}]$^21^
Specific Heat, $C$	3540 $[{\text{J}}/{\text{kg}}.{}^{ \circ }{\text{C}}]$^21^	3540 $[{\text{J}}/{\text{kg}}.{}^{ \circ }{\text{C}}]$^21^
Absorption Coefficient, $\sigma_{a}$	2 $[1/{\text{m}}]$^22^	6 $[1/{\text{m}}]$^22^
Scattering Coefficient, $\sigma_{s}$	65 $[1/{\text{m}}]$^22^	5 $[1/{\text{m}}]$^22^
Electric Conductivity, $\sigma$	1.69 $[{\text{S}}/{\text{m}}]$^23^	1.69 $[{\text{S}}/{\text{m}}]$^23^
Relative Permittivity, $\varepsilon_{r}$	43.03^23^	43.03^23^
Relative Permeability, $\mu_{r}$	1^23^	1^23^
Metabolic Heat Source, $Q_{m}$	33,800 $[{\text{W}}/{\text{m}}^{3} ]$^24^	33,800 $[{\text{W}}/{\text{m}}^{3} ]$^24^
Blood Perfusion, $\omega_{b}$	3.6 × $10^{ - 3} [1/{\text{s}}]$^24^	3.6 × $10^{ - 3} [1/{\text{s}}]$^21^
Activation Energy, $dE$	2.577 × $10^{5}$ $[{\text{J}}/{\text{mol}}]$^21^	2.577 × $10^{5}$ $[{\text{J}}/{\text{mol}}]$^21^
Frequency Factor, $A$	7.39 × $10^{39}$ $[1/{\text{s}}]$^21^	7.39 × $10^{39}$ $[1/{\text{s}}]$^21^

**Table 2:** Thermal features of blood^24^.

Property	Value
Density of blood, $\rho_{b}$	1060 $[{\text{kg}}/{\text{m}}^{3} ]$
Specific heat capacity of blood, $C_{b}$	3600 $[{\text{J}}/{\text{kg}}.{}^{ \circ }{\text{C}}]$
Temperature of blood, $T_{b}$	$37^{ \circ } {\text{C}}$

It should be mentioned that here the equations are solved by finite element method (FEM) in Matlab software and due to the large number of elements is three dimensions and limitations of the solver for solving such models, it is assumed that the model has the axial symmetry. Therefore, the problem is solved in 2D-axisymmetric cross section. The microwave antenna is inserted into the tumor from the bottom, while the laser beam irradiates on the top surface of the tumor. The antenna used in this research is a coaxial single-slot antenna which is a thin coaxial cable with a ring-shaped slot on the outer conductor. One of the advantages of this type of microwave antenna is that the size of coagulated region perpendicular to the antenna is more in coaxial-slot antenna compared to other types. Here, the selected antenna operates at 2.45 GHz, which is a frequency widely used for coagulation therapy^23,24^. The structure of antenna is depicted in Fig. 1. The geometrical dimensions and physical properties of the antenna are given in Table 3.

**Table 3:** Dimensional and electromagnetic properties of microwave antenna for Hepatocellular carcinoma treatment^23^.

Property	Value
Diameter of the central conductor	0.29 mm
Diameter of catheter	1.79 mm
Inner diameter of the outer conductor	0.94 mm
Outer diameter of the outer conductor	1.19 mm
Relative permittivity of catheter, $\varepsilon_{cat}$	2.6
Relative permittivity of inner dielectric, $\varepsilon_{diel}$	2.03

In this model, three lasers with the same wavelength of 796 nm and different powers of 2 W/cm^2^ (laser 1), 0.5 W/cm^2^ (laser 2) and 0.25 W/cm^2^ (laser 3) are used for liver cancer treatment. As shown in Fig. 1, the spot size of the first laser is twice the other two lasers. The reason of all these differences is that in superficial regions of the tumor, more accurate lasers with less powers are needed for avoiding side effects. It should be stressed that in the real experiment, one may use a unique laser several times for coagulation of all the edges of tumor with top accuracy, while the bulk of the tumor can be coagulated by microwave radiation. The laser used here is a pulsed laser with the exposure time of 50 s and cooling time of 20 s. Employing pulsed laser instead of continuous laser can help us to coagulate the desired region without overheating the surrounding tissues^25,26^.

Gold naorods (GNRs) with a 5 nm diameter and an aspect ratio of 3.5 (e.g. length of 17–18 nm) are injected into the tumorous region to enhance the heating absorption. The volume fraction of GNRs is assumed to be 0.001%. The absorption and scattering coefficient of proposed nanorods with the given volume fraction at 796 nm is 121/cm and 0.5/cm respectively^22^.

### Theory

The main challenge associated with the physical bioheat problems, which will be discussed in current research, is evaluation of the temperature profile and the fluence rate distribution in the tumorous region with knowing the governing initial and boundary conditions, geometry, heat sources, as well as the thermophysical and optical properties of the tissues. In living tissues, blood perfusion and passage of blood modifies the heat transfer. Furthermore, metabolic activity generates heat within the tissue. Therefore, an equation is needed for describing the heat transfer in tissue by considering the effects of both blood perfusion and metabolism. This relation, widely known as Pennes equation, was first established by Penne (1948) and Perl (1962)^2,27,28^:

1 $$ \rho C_{P} \frac{\partial T}{{\partial t}} = \nabla (k\nabla T) - \rho_{b} \omega_{b} C_{b} (T - T_{b} ) + Q_{met} + Q_{S} $$

where, $\rho_{b}$ is the blood density $({\text{kgm}}^{ - 3} )$, $C_{b}$ is the heat capacity of blood $({\text{Jkg}}^{ - 1} \;^{ \circ } {\text{C}}^{ - 1} )$, $k$ is the thermal conductivity of the tissue $({\text{Wm}}^{ - 1} \;^{ \circ } {\text{C}}^{ - 1} )$, $T$ is the temperature $(^{ \circ } {\text{C}})$,$\omega_{b}$ is the blood perfusion $( {\text{ml}} \cdot {\text{S}}^{ - 1} {\text{cm}}^{ - 3} )$, *ρ* is the tissue density, $T_{b}$ is the body core temperature $(^{ \circ } {\text{C}})$, $Q_{met}$ indicates the metabolic heat source term $({\text{Wm}}^{ - 3} )$ and $Q_{S}$ is the external heat source. In our models there are two heat sources; microwaves and laser. So, $Q_{S} = Q_{MW} + Q_{laser}$. Each of these heat sources will be analyzed individually and then, will insert into Eq. (1). In this research, due to no variation in the laser frequency, the dispersion models are not applied.

### Heating by microwave irradiation

Tumor treating by the electric field of microwave antenna is known as a noninvasive, regional antimitotic treatment modality for the treatment of various diseases such as glioblastoma^29^, breast^30,31^, liver^32,33^, skin^34^, and prostate^35^ cancer. Generally, microwave energy is converted into heat within the tissue due to dielectric losses^36^. Since microwave wavelengths in tissue are in the cm order, their propagation and absorption in tissue is governed by Maxwell’s equations as below:

2 $$ \nabla \cdot D = \rho $$

3 $$ \nabla \cdot B = 0 $$

4 $$ \nabla \times E = - \frac{\partial B}{{\partial t}} $$

5 $$ \nabla \times H = J + \frac{\partial D}{{\partial t}} $$

here, $D\;[{\text{c}}/{\text{m}}^{2} ]$ is the electric flux density, $B\;[{\text{T}}]$ is magnetic field, $E\;[{\text{V}}/{\text{m}}]$ is electric field strength, $H\;[{\text{A}}/{\text{m}}]$ is magnetic field intensity, $\rho \;[{\text{C}}/{\text{m}}^{2} ]$ is free charge density, and $J\;[{\text{A}}/{\text{m}}^{2} ]$ is current density.

In microwave coagulation therapy, a thin microwave antenna is inserted into the tumor. Propagation of electromagnetic waves into the tissue has two steps; (i) their propagation in the dielectric of coaxial cable, and (ii) their propagation and dissipation in the tissue. In the coaxial cable, the electromagnetic wave propagation is characterized by transverse electromagnetic fields (TEM), for which the following equations are held in time domain ^37,38^:

6 $$ E = \hat{r}\frac{C}{r}e^{{j\left( {\omega t - kz} \right)}} $$

7 $$ H = \hat{\varphi }\frac{C}{rZ}e^{{j\left( {\omega t - kz} \right)}} , $$

where, $r,\varphi$ and z are cylindrical coordinates centered on the axis of the coaxial cable,$\omega$ is the angular frequency and $Z$ is the wave impedance in the dielectric part of the cable. The magnitude and direction of electromagnetic energy transfer is given by the Poynting vector *S*. The time average of Poynting vector is $\overline{S} = \frac{1}{2}{\text{Re}} \left( {E \times H^{*} } \right)$. Hence, for finding the in the cable, one can use this relation^37^:

8 $$ P = \int_{{r_{in} }}^{{r_{out} }} {{\text{Re}} \left( {\frac{1}{2}E \times H^{*} } \right)2\pi rdr = \hat{z}\pi \frac{{C^{2} }}{Z}\ln \left( {\frac{{r_{out} }}{{r_{in} }}} \right)} , $$

In this equation, $r_{in}$ and $r_{out}$ refer to the inner and outer radii of the coaxial cable, respectively. By knowing the average power in the cable, *C* can be derived and, so, *E* and $H$ can be easily calculated at all points.

In the tissue, magnetic field has only an azimuthal component so, it can be modeled as an axisymmetric transverse magnetic (TM) field. The scalar $H_{\varphi }$ for TM field is given by below equation as^37^:

9 $$ \nabla \times \left( {\left( {\varepsilon_{r} - j\frac{\sigma }{{\omega \varepsilon_{0} }}} \right)^{ - 1} \nabla \times H_{\varphi } } \right) - \mu_{r} k_{0}^{2} H_{\varphi } = 0, $$

here, $\sigma [{\text{S}}/{\text{m}}]$ is the electrical conductivity of the tissue. Taking into account the boundary condition on metallic surfaces ($\hat{n} \times E = 0$) and Eqs. (6) to (9), $E$ and $H$ can be determined at all points.

The energy from microwaves, which serves as heat source $Q_{MW}$ in the heat transfer equation, is given by the below equation as^37^:

10 $$ Q_{MW} = \frac{\sigma }{2}\left| E \right|^{2} . $$

If nanoparticles are added to the tissue, their effect on the electromagnetic properties of the medium must be considered too. Gold is one of the non-ferromagnetic metals with the permeability near to one. So, the amount of $\mu_{r}$ for the tissue remains unchanged by adding GNPs. Besides, according to Ostovari et.al.^39^ at frequencies above10 KHz, addition of gold nanoparticles to the tissue doesn’t change the electrical properties (electrical conductivity and permittivity) of the tissue. Usually, Au NPs injected to the tissue are covered by ions and other charged particles and so, they can be considered as electrical dipole moments. Like polar molecules, Au NPs can affect displacement current and capacitive impedance, but as the frequency increases, reorientation of these dipoles with alternative current electric field becomes more difficult and their influence on displacement current enhancement will be restricted.

Absorption of microwaves in tissue is not considerably affected by Au NPs. Because the range of wavelengths where the nanoparticles’ absorption has dramatic increase is in nm range^40–42^, far less than the microwaves’ wavelength in this research. So, generally at the frequency of 2.45 GHz, presence of Au NPs has no considerable effect neither on the electromagnetic field of microwaves in the tissue nor on their absorption.

### Heating due to the photothermal effect of laser irradiation

The laser beam illumination can be considered as a heat source in the electromagnetic calculation. When an incident laser beam passes through a medium in direction Ω, it interacts with the medium. Part of its intensity is absorbed and other fractions are scattered in another direction of $\Omega^{\prime }$. The fraction that is absorbed is shown by $\sigma_{a} I\left( \Omega \right)$ and the scattered fraction is indicated by $\sigma_{s} I\left( \Omega \right)$, where $\sigma_{a} [1/m]$ and $\sigma_{s} [1/m]$ are absorption and scattering coefficients. For obtaining the energy deposited at each point, one needs to solve the radiative transfer equation (RTE)^43^. For quasi-static conditions, which is satisfied for the present study, RTE obeys the following equation:

11 $$ \Omega \cdot \nabla I\left( \Omega \right) = \sigma_{a} I_{b} \left( T \right) - \left( {\sigma_{a} + \sigma_{s} } \right)I\left( \Omega \right) + \frac{{\sigma_{s} }}{4\pi }\int_{4\pi } {I\left( {\Omega^{\prime } } \right)\phi \left( {\Omega^{\prime } ,\Omega } \right)d\Omega^{\prime } } . $$

In the above equation, $I_{b}$ is the blackbody intensity given by the Plank function and $\phi \left( {\Omega^{\prime } ,\Omega } \right)$ is the probability that a ray from direction $\Omega^{\prime }$ scatters into direction $\Omega$. The last term of the right-hand side of this equation accounts for the portion of the radiative energy coming from all possible directions which is scattered toward the considered direction of propagation.

There are different methods for solving this equation. One of these methods is P1-approximation, which is a first order approximation of spherical harmonics of radiation field. This method is simple and computationally cheap, however, it is proved to produce reasonable results in simulations of GNP-mediated photothermal therapy^44^. According to Modest and Tabanfar’s calculation^45^ in P1-approximation if incident radiation $G$ is defined as:

12 $$ G = \int\limits_{4\pi } {I\left( \Omega \right)d\Omega } , $$

and radiative heat flux crossing an element is given by^45^:

13 $$ q_{r} = \int\limits_{4\pi } {I\left( \Omega \right)\Omega d\Omega } , $$

Then, the RTE equation can be rewritten in terms of $G$ and $q_{r}$ as^45^:

14 $$ I\left( {r,\hat{\Omega }} \right) = \frac{1}{4\pi }\left[ {G\left( r \right) + 3q\left( r \right) \cdot \hat{\Omega }} \right]. $$

When the medium is irradiated by laser, the term *I* in this equation has two components; one collimated beam, which is the external emission coming from the outside, and another the diffusion term that arises due to scattering. According to Modest and Tabanfar explanation, in such problem, the effect of external radiation field can be considered by adding a source term to the diffusion relations. So, RTE will be reduced to a simple diffusion relation as^45^:

15 $$ \nabla \cdot \left( {D_{P1} \nabla G} \right) - \sigma_{a} \left( {G - 4\pi I_{b} } \right) = 0, $$

where, $D_{P1}$ is the P1 diffusion coefficient defined as^45^:

16 $$ D_{P1} = \frac{1}{{3\sigma_{a} + \sigma_{{s\left( {3 - a_{1} } \right)}} }}. $$

$a_{1}$ is the linear Legendre coefficient. This coefficient relates to anisotropy so that for the case of isotropic scattering $a_{1}$ equals to zero.

The term related to the laser heat source in Penne equation, $Q_{laser}$ is calculated by the divergence of the radiative heat flux, $q_{r}$. That is because the radiative heat flux crossing an element with an area normal to the direction of $\Omega$ is a result of intensities incident from all directions and it is the divergence of heat flux at any point which leads to rising the temperature at that point. It should be stressed that once $G$ is characterized at each point, $\nabla \cdot q_{r}$ can be found.

Moreover, addition of gold nanoparticles significantly modifies the optical features of the tissue. Several studies have shown that when laser excites gold nanorods^41,46^ and nanoshells^40,42^ depending on the nanoparticle’s geometrical properties, they will have peaks in their plasmon’s wavelengths. For instance, Soni et al.^41^ showed that the GNRs with 5 nm diameter and an aspect ratio of 3.5 have a peak in plasmon’s wavelength at 796 nm. According to this paper, at the laser wavelength of 796 nm, optical absorption and scattering coefficient for GNR concentration of 0.001% are $121/{\text{cm}}$ and $0.5/{\text{cm}}$, respectively.

The preference of infrared and near-infrared laser-induced thermal therapy (LITT) is attributed to the exact controllability^47,48^ and appropriate ‘optical window’ of tissue^49^ in this wavelength region.

### Thermal damage

An important concern in research about bioheat transfer is accurate and appropriate thermal damage of the diseased tissues without any destruction of the neighboring healthy tissues during tumor treatment. Several investigations have demonstrated that tissue damage depends on both of the temperature and exposure time. It should be mentioned that as tissue temperature increases, the elapsed time necessary to achieve the threshold of damages decreases. The progression of thermal injury can be reasonably approximated by Arrhenius equation. This relation is usually utilized for illustrating the rate of the irreversible heating damage of the biological tissues^26,50^:

17 $$ \Omega (t) = \int_{0}^{t} {A.e^{{\frac{ - \Delta E}{{RT}}}} } dt, $$

where, $\Omega (t)$ indicates the degree of tissue injury, $R$ is the universal gas constant,$A$ is the frequency factor for kinetic expression $({\text{s}}^{ - 1} )$, and $\Delta E$ is the activation energy for irreversible damage reaction $({\text{J}} \cdot {\text{mol}}^{ - 1} )$. The critical value of $\Omega = 1$ is the point when thermal necrosis occurs. The parameters *A* and $\Delta E$ depend on the type of tissue, which are presented for the liver in Table 1.

### Validation of the simulation results

**Figure 2:**
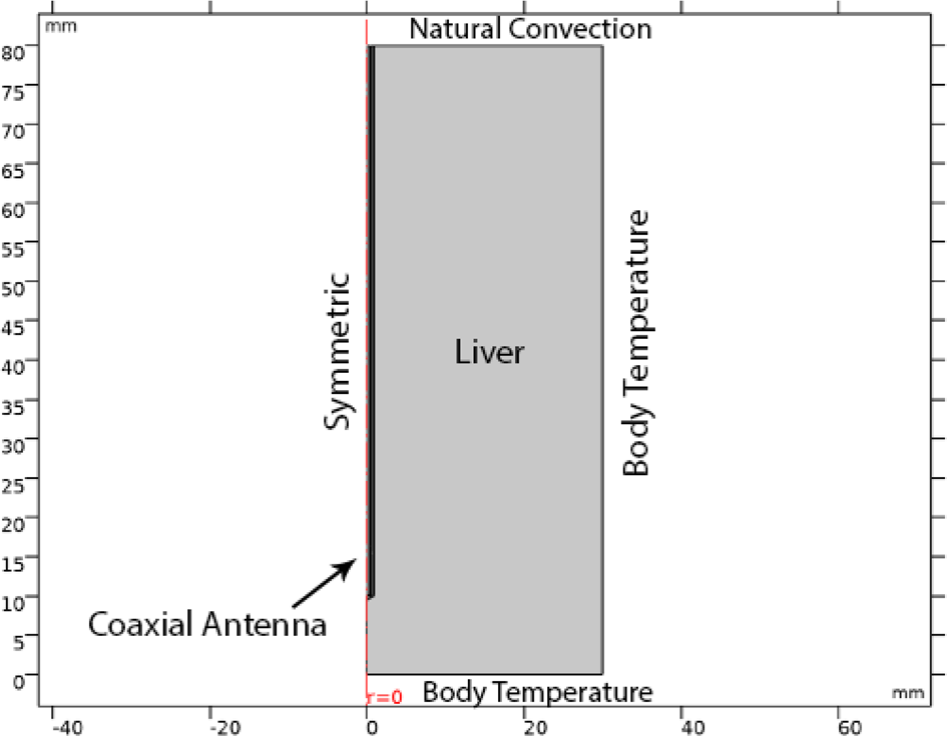
The geometry of the model produced by Saito et al.^23^, which is used for validation of the data of simulation during the microwave antenna irradiation. Reprinted with permission from^22^.

**Figure 3:**
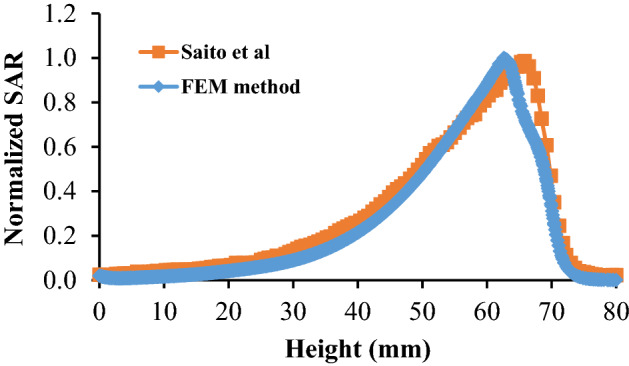
A comparison between SAR quantity calculated by Saito et al.^23^, and present model by FEM method.

To validate the accuracy of the proposed model, the results of RF antenna simulation and nanoparticle-mediated laser therapy model are compared with previous literatures. For instance, Saito et al. in 2001 have estimated the heating characteristics of a special type of microwave antenna with a coaxial slot using finite element method^23^. To check accuracy of our model, the same simulation is performed with similar dimensions and boundary conditions as they utilized. Figure 2 shows the 2D axisymmetric geometry of the model produced by Saito et al. The tip of the antenna has a slot, just the same as the antenna depicted in Fig. 1. All the physical properties used in their problem are exactly the same as the features mentioned in Tables 1, 2 and 3, except that they didn’t consider the differences of some physical parameters between tumor and healthy tissue. Figure 3 presents a comparison between this paper’ data for SAR (specific absorption coefficient) distribution at a distance of 2.5 mm from the antenna axis with the data of Saito’s reports. As can be clearly seen in this figure, the data of two models are in good agreement with each other. It should be mentioned that SAR is defined as the ratio of absorbed heat power to the tissue’s density.

**Figure 4:**
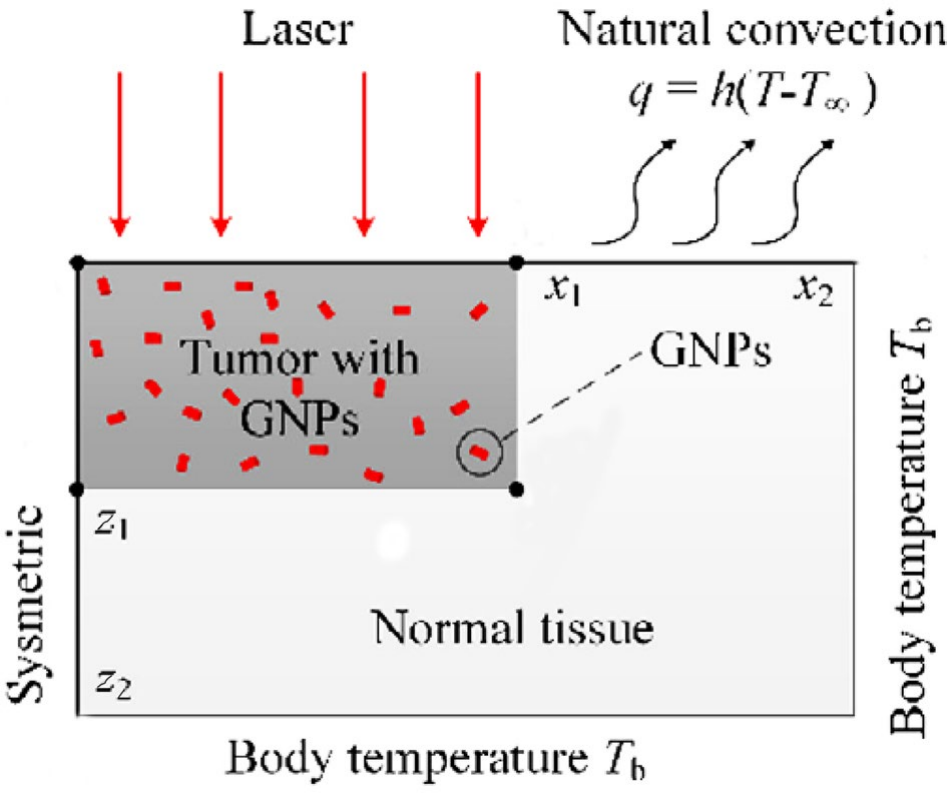
The geometry of the model produced by Sonie et al.^22^ for validation of the simulation data of nanoparticle-mediated photothermal therapy. Reprinted with permission from^22^.

Furthermore, Sonie et al.^22^ in 2014 have investigated the effects of nanoparticles injection in thermal ablation of skin tumors. Figure 4 is the schematic of the problem they have solved. In this figure, the skin tissue dimensions were as: $x_{1} = 10\;{\text{mm}}$, $x_{2} = 20\;{\text{mm}}$, $z_{1} = 5\;{\text{mm}}$ and $z_{2} = 10\;{\text{mm}}$. The incident laser intensity was selected as $0.5\;{\text{W}}/{\text{cm}}^{2}$. The physical properties of the skin tissue that Sonie et al. have modeled are represented in Table 4. The value of absorption coefficient and scattering coefficient of the tissue are the same as the magnitudes in Table 1.

**Table 4:** Physical properties of skin (used by Sonie et al.^21^).

Property	Value
Density of healthy tissue	$1000\;{\text{kg}}/{\text{m}}^{3}$
Density of tumor	$1100\;{\text{kg}}/{\text{m}}^{3}$
Thermal conductivity	$0.52\;{\text{W}}/{\text{m}}.^{ \circ } {\text{k}}$
Specific Heat	$4200\;{\text{J}}/{\text{kg}}.^{ \circ } {\text{k}}$
The metabolic heat source	$1091\;{\text{W}}/{\text{m}}^{3}$
Blood perfusion rate	$9.1 \times 10^{ - 4} /{\text{s}}$

To validate the results of this simulation in the section of nanoparticle-mediated photothermal therapy, a comparison is performed with a simulation represented by Sonie et al. including similar geometry and physical properties as the current work. It should be stressed that all the boundary conditions of these two models are also the same. The only difference between these two models is that they used Beer Lambert law for evaluation of the absorption of laser irradiation by the nanoparticles, while here, the P1-approximation method is used for estimation of the temperature. Generally, Beer Lambert law is a simplification of RTE equation which neglects the refraction, reflection, or scattering of beams in the medium. It can give satisfactory results for most absorbing-scattering media, however, its accuracy is less than P1-approximation, which takes into account the isotropic and linear anisotropic scattering.

**Figure 5:**
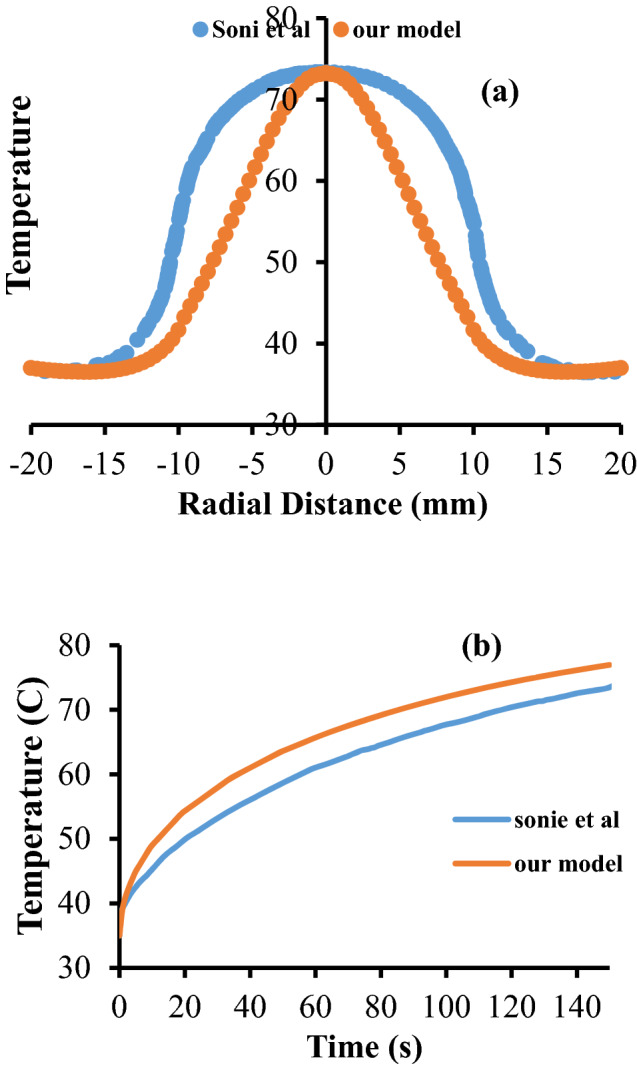
Comparison of the temperature calculated by Sonie et al.^22^ and P1-approximation model related to this paper.

Figure 5 shows a comparison between the temperature achieved by two models of Sonie et al. and P1-approximation which illustrates a relatively good agreement in two graphs in regards of trend and maximum. Mismatch of two graphs in some points can be attributed to the differences of the computational methods in approximating RTE equation.

## Results and discussions

In this paper, the influences of microwave ablation through a single-slot coaxial antenna and laser irradiation on the Hepatocellular carcinoma tissue is investigated. Usually it is hard to eradicate the tumor area without any damage to the surrounding healthy tissues. Hence, for minimizing the risk of thermal tissue destruction, gold nanoparticles are injected into the tumor, under irradiation of a diode laser, to enhance the tumor temperature beyond the normal physiological level accompanied by protecting the surrounding healthy tissues. It should be mentioned that the lasers utilized here are pulsed lasers with the heating time of 50 s and cooling time of 20 s. To figure out the effects of gold nanoparticles on the enhancement of temperature in the tissue, the problem is solved once with and again without insertion of Au nanoparticles and then, the comparison’s results are presented.

### The effect of gold nanoparticles injection

In this section, the influence of nanoparticles injection on the temperature rise of the tumor is investigated. In section 2-1, according to the previous researches^39^ it was mentioned that during irradiation of microwave antenna on gold nanoparticles, at frequencies above 10 kHz, the addition of gold nanoparticles to the tissue didn’t change the electrical properties (electrical conductivity and permittivity) of the tissue. Hence, it can be concluded that the temperature will not change significantly. So, in this section, only the effect of nanoparticles on photothermal heating of the tissue is considered.

**Figure 6:**
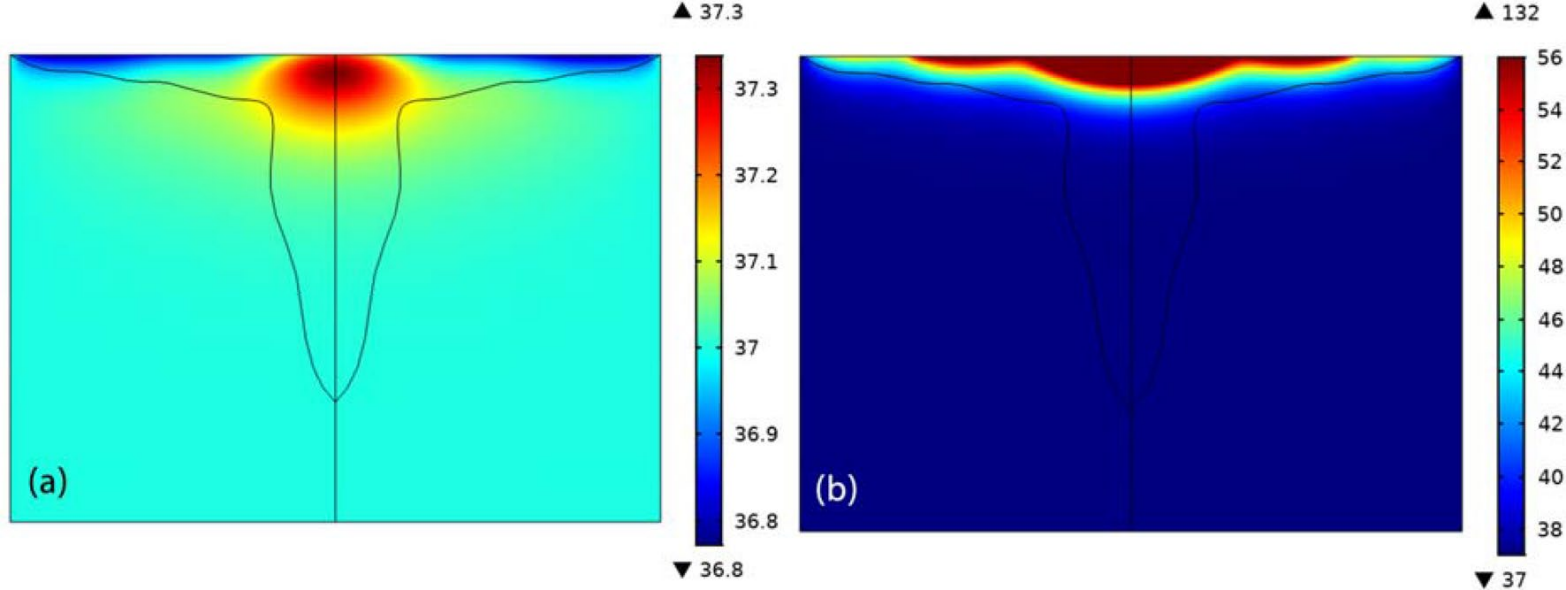
Temperature of the tissue after 10 min of laser irradiation in the absence (left) and presence (right) of GNPs.

Figure 6 Temperature of the tissue after 10 min of laser irradiation in the absence (left) and presence (right) of GNPs. As can be seen in this figure, in the presence of nanoparticles, the temperature is raised to $132^{ \circ } {\text{C}}$ on the surface, while in the absence of nanoparticles the temperature of the tissue doesn’t exceed $37.3^{ \circ } {\text{C}}$, even in superficial regions. Likewise, in the absence of GNPs, the fraction of necrosis in each part of the tumor is not more than 0.17%. It means that the tumor remains totally intact, but in the presence of GNPs, in 35% of the tumor region, the fraction of necrotic tissue is more than 75%. It can be attributed to plasmon resonance of gold nanorods during of laser illumination which increase the tissue’s temperature greatly.

**Figure 7:**
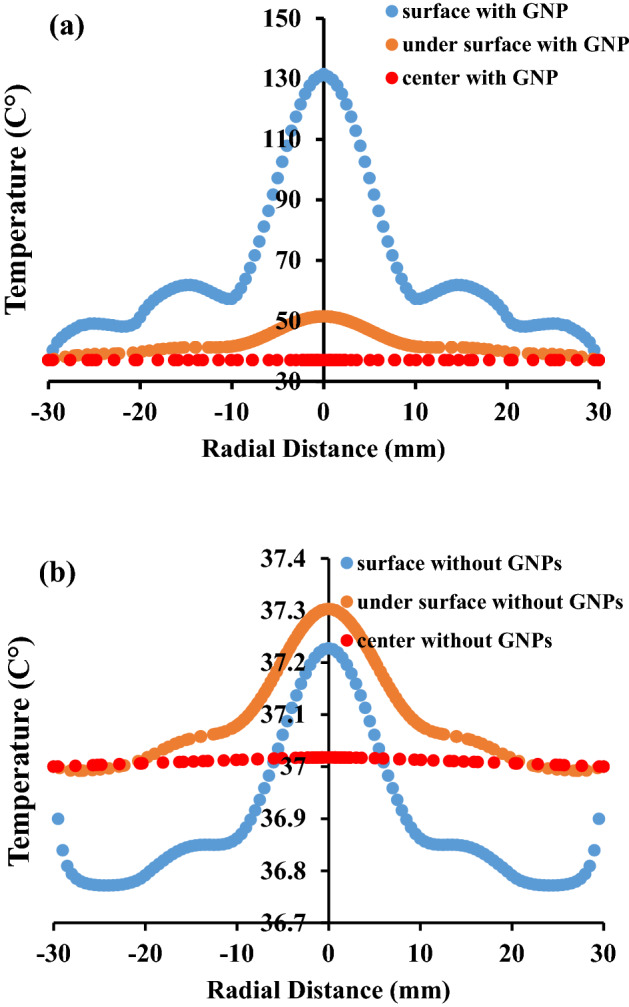
Temperature of the tumor along three lines in different depths of the tumor and a comparison of the temperature (**a**) with and (**b**) without any GNPs.

For better understanding the effects of nanoparticle injection, the tissue’s temperature is estimated at three different depths of tumor: (i) line 1 which is located at tumor surface (z = 43 mm), (ii) line 2 which is related to three millimeters below the surface (z = 40 mm), and (iii) line 3 which crosses the tumor’s center (z = 27 mm). Figure 7 represents a comparison between the tissue’s temperature along these three lines. The surface temperature clearly shows that we have used three lasers in 2D axisymmetric model, which resembles implementing five lasers in 2D cross section, just like what you can see in Fig. 1. It should be mentioned that the power of the central laser is twice other two lasers.

In the absence of GNPs, the temperature of the points near to the tissue’s surface, like the points in line 2 i.e. 3 mm below the surface, is even lower than the body temperature. That is due to the fact that in these points, the rate of heating is less than the rate of heat loss which happens because of normal conduction. Another noteworthy point about these graphs is that even when GNPs are injected into the tissue, laser is not able to coagulate deep parts of the tumor, like the center. This is why using microwave antenna is proposed for coagulation of deep tumors, in parallel with laser therapy.

### Simultaneous irradiation of microwave and laser beam with injection of gold nanoparticles

**Figure 8:**
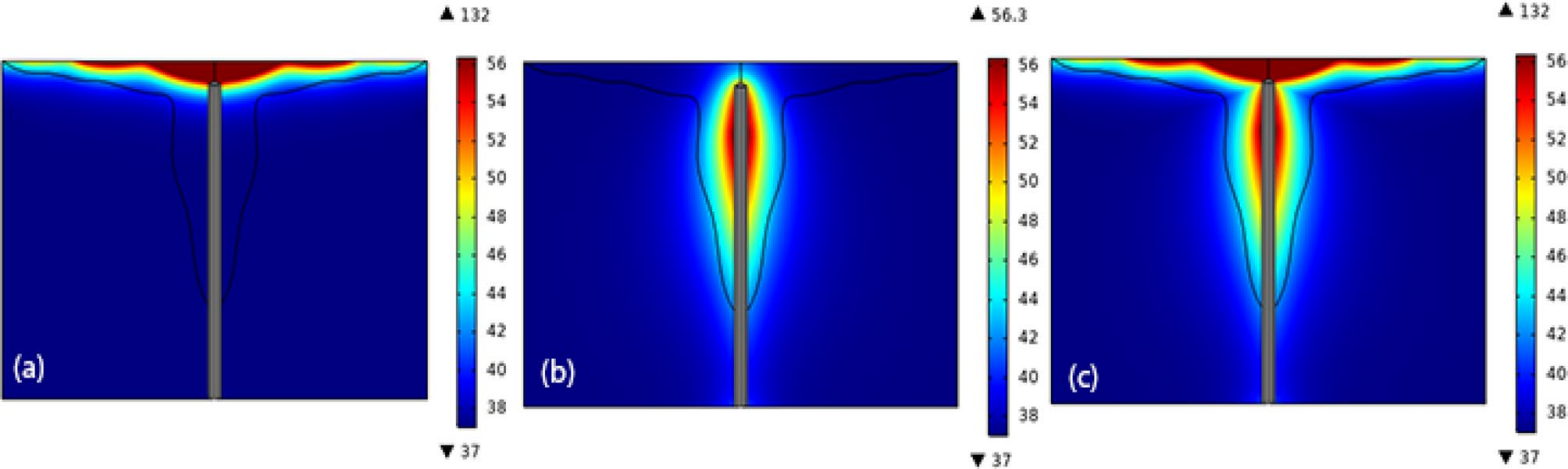
Temperature (degrees of Celsius) of the tumor in the presence of gold nanoparticles when (**a**) only pulsed laser is irradiated, (**b**) only microwave antenna is implemented, and (**c**) when both are utilized.

In this section, the microwave antenna is added to the model as shown in Fig. 8 and it is followed how effective can be for coagulation of the tumors in deep sections of tumor. Here, all the laser beams and the microwave antenna are irradiated for 10 min. As it is seen in all of three cases, the minimum temperature is $37^{ \circ } {\text{C}}$, i.e. the body temperature. Moreover, the maximum temperature achieved by the microwave antenna (Fig. 8a) is $56^{ \circ } {\text{C}}$, while laser can increase the temperature of the tissue up to $132^{ \circ } {\text{C}}$ on the surface. It should be stressed that in both Fig. 8a and c, the areas with temperatures higher than $56^{ \circ } {\text{C}}$ are shown by dark red.

**Figure 9:**
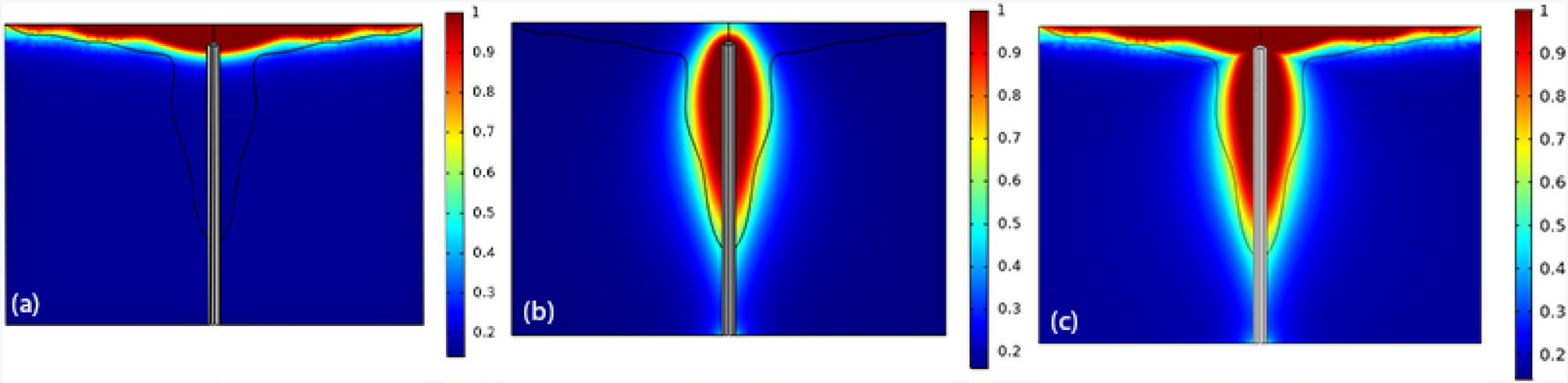
The fraction of damage of the tissue in the presence of gold nanoparticles when (**a**) the microwave antenna is implemented for 10 min, (**b**) pulsed laser is irradiated for 10 min, and (**c**) when both are irradiated for 10 min.

The fraction of the tissue’s damage is the main parameter which determines how successful the method can be and is presented in Fig. 9. Figure 9a shows the fraction of the damage after 10 min of laser illumination. Furthermore, Fig. 9b presents the fraction of damage after 10 min irradiation of microwave antenna. Finally, Fig. 9c illustrates the status when the tissue is simultaneously irradiated by both of laser and microwave antenna for 10 min.

**Figure 10:**
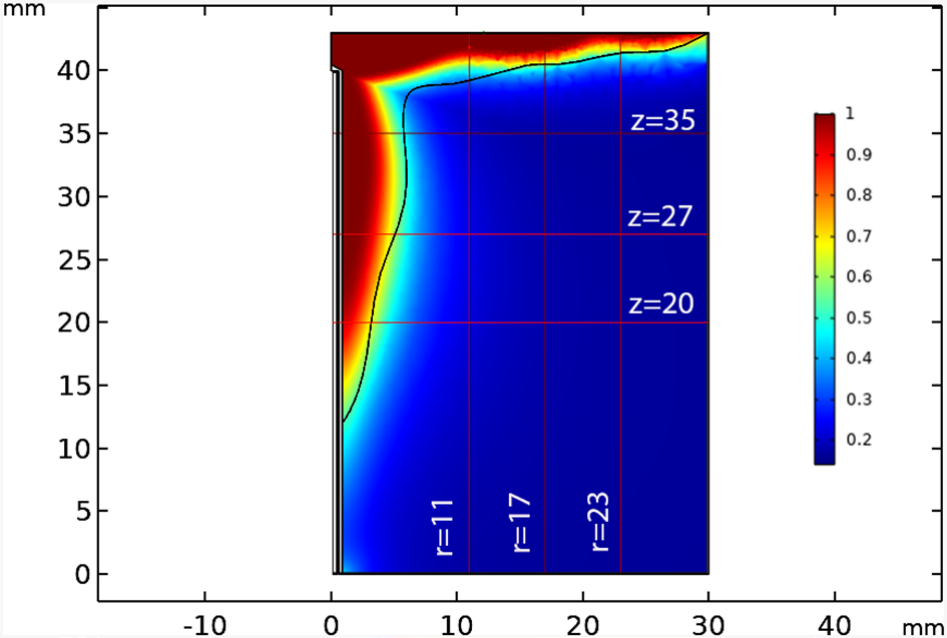
Six horizontal and vertical lines for estimation of the amount of damage to the neighbor areas of tumor.

**Figure 11:**
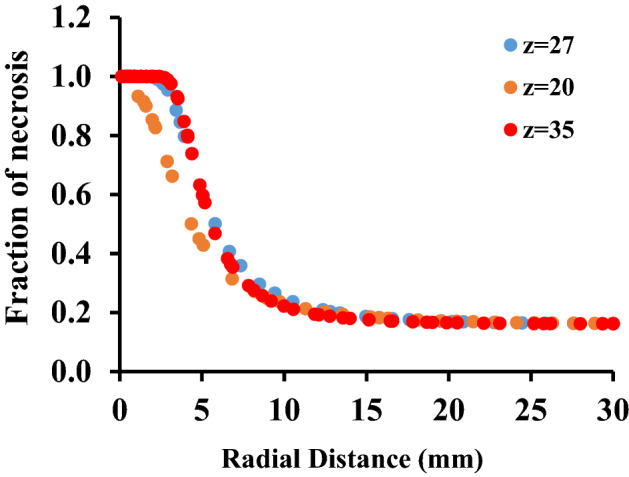
The fraction of tissue’s necrosis versus radial distances at constant horizontal positions of z = 20, 27 and 35 mm.

**Figure 12:**
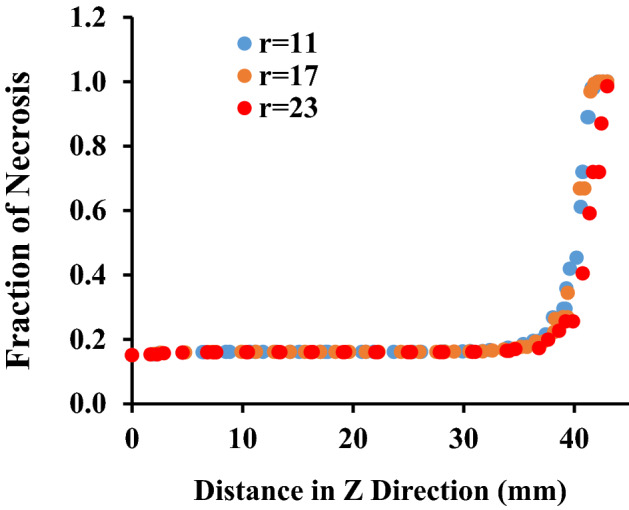
The fraction of tissue’s necrosis along different horizontal lines, at fixed radial distances of r = 11, 17 and 23 mm.

If the areas where the fraction of necrotic tissue is more than 0.75 are considered as the necrosis tissue, the results of image processing show that more than 72 percent of the tumor occur to necrosis by simultaneous usage of laser and microwave irradiation. It must be mentioned that while laser or microwave antenna are implemented alone, just 35% and 37% of the tumor encounter necrosis, respectively. Here, to check whether the proposed method has damaged the surrounding healthy tissue or not, the fraction of the necrotic tissue along six lines, crossing the tissue model is estimated at different places. Figure 10 shows the position of these selected six lines in the liver tumor for evaluating the side effects of the proposed method. Choosing three vertical lines and three horizontal lines helps us to have a better judgment on the damage estimation in both of superficial sections and deep areas. For more clarity, Figs. 11 and 12 show the fraction of necrosis along the horizontal and vertical lines, respectively. The horizontal lines are located at z = 20, z = 27. and z = 35. The vertical lines are located at r = 11, r = 17 and r = 23. According to Fig. 10, the yellow color (i.e. the necrosis fraction equals to 0.7) presents the border of safe region which illustrates that there is no severe damage in normal areas of tissues.

As it can be seen clearly in Fig. 11, at z = 35 and in lower radial distances, wider regions are exposed to necrosis due to being at neighborhood of laser and microwave irradiations. Furthermore, at far radial distances in all of horizontal lines, the necrosis’s fraction is low which illustrates the absence of the side effects in this treatment method.

In Fig. 12, similar behavior is observed at different radial distances, with a little increase at r = 11 mm. Moreover, as can be clearly seen in this figure, at z > 42 mm, the necrosis fraction is beyond of 0.7 which presents that just tumor areas are exposed to damage and affirms the helpfulness of the proposed technique.

## Conclusion

In this research, the laser-liver interaction is studied in the presence of gold nanoparticles and the effectiveness of combining this method with microwave therapy is also investigated. The results of FEM (finite element method) simulations presented that this simultaneous therapy can be exceptionally beneficial for treatment of the tumors including both superficial and deep parts. Here, the heat generated by the microwave antenna was calculated by estimating the amount of electromagnetic energy loss in the tissue. Furthermore, the magnitude of energy produced by laser irradiation and absorbed by GNPs was found by solving the RTE equation. For solving RTE equation, P1-approximation was used which means that both of isotropic and linear anisotropic scatterings were considered. Finally, Pennes’ equation was solved to find the temperature inside of the tumor. In addition, the results of simulations of each step were successfully validated with the results of previous literatures.

In summary, the results showed that; (i) Gold nanoparticle injection greatly enhanced the effectiveness of photothermal therapy. (ii) Without nanoparticles injection, 10 min of laser irradiation couldn’t raise the temperature more than $37.3^{ \circ } {\text{C}}$. (iii) Moreover, after nanoparticles injection, in the same conditions, the temperature could increase up to $132^{ \circ } {\text{C}}$ and the process of necrosis got started in the tumor. (iv) After 10 min of simultaneous laser and microwave irradiations, the fraction of necrotic tissue in more than 72 percent of the tumor areas exceeded 0.75, (v) while during implementing the laser or microwave alone, the necrosis fraction was just about 35% and 37%, respectively. (vi) Moreover, the side effects or the amount of damage to the surrounding healthy tissue was negligible. So, the proposed combinational irradiation of laser and microwave antenna can enhance the coagulation therapy of complicated tumors dramatically with nearly no damage to the surrounding area, if the proper powers are chosen.
